# Empirical investigation of the effect of adding nanoparticles to HB-80 gas turbine oil: Evaluation of thermophysical behaviors

**DOI:** 10.1016/j.heliyon.2024.e29759

**Published:** 2024-04-17

**Authors:** Shahram Karimi, Amir Homayoon Meghdadi Isfahani, Masoud Afrand, Mohammad Akbari

**Affiliations:** a Department of Mechanical Engineering, Majlesi Branch, Islamic Azad University, Isfahan, Iran; b Department of Mechanical Engineering, Najafabad Branch, Islamic Azad University, Najafabad, Iran; c Aerospace and Energy Conversion Research Center, Najafabad Branch, Islamic Azad University, Najafabad, Iran

**Keywords:** Nano lubricant, Thermal conductivity, Wear, Viscosity, Flash point

## Abstract

The current study aims to conduct an empirical investigation on the performance of copper (II) oxide (CuO), Graphene Oxide (GO), and Molybdenum (VI) Oxide (MoO3) nanoparticles as additives for HB-80 base lubricant and their effects on essential characteristics such as density, flashpoint and flammability, cloud point and pour point, viscosity and viscosity indicators, thermal conductivity coefficient, friction coefficient and wear. The test results show that nanoparticles have minor effects on density and viscosity but managed to improve viscosity indicators. Flashpoint, flammability, cloud point, and pour point all increased after the addition of nanoparticles. The increase in the concentration of nanoparticles also improved and increased the lubricant's thermal conductivity coefficient; the highest increase was 11.3 % compared to the base oil with the addition of 1 wt% CuO. Adding nanoparticles to lubricant decreases wear and friction coefficient by creating a lubricant film, especially at lower nanoparticle concentrations, which leads to a more stable lubricant film by nanoparticles. Copper (II) Oxide nanoparticles had the best performance in reducing friction coefficient and wear at 0.2 wt% with 22.86 % and 63.92 % reduction compared to the base oil, respectively.

## 1 Introduction

Today, lubricants have widespread use in industrial processes. Lubricants are materials placed between two surfaces to create a protective layer and prevent direct contact between these two surfaces, thus reducing friction and wear and removing the heat and particles created due to wear [[1], [2], [3]].

In mechanical instruments such as turbines and gas compressors, friction is one of the main reasons for energy loss. Cooling of gas turbines is also a major problem, especially in hot environments. Significant increase in turbine temperature can damage turbine fins and bearings, thus reducing their effective life. Usually in industrial conditions, it is advised to reduce turbine power at higher environment temperatures in order to prevent these problems. Therefore, turbine oil performs two major tasks at the same time: 1- cooling turbine parts 2- reducing friction and lubrication.

Improving the thermal conductivity of the lubricant oil by adding nanoparticles means that a lower flow of lubricant can remove a higher amount of heat from turbine which in turn reduces the necessary pumping power (input work) and electrical power in cooling fans used to reduce lubricant temperature. Furthermore, addition of nanoparticles in lubricants can significantly reduce friction between moving parts of the turbine or compressor which results in lower damage and maintenance costs in long-term.

Since HB-80 lubricant oil is used often in gas turbines, this study used it as the bases for the tests. In addition to gas turbines, HB 80 oil is used in steam turbines, high-speed industrial gears and special industrial bearings.

Nanotechnology has affected many industries (such as heat transfer [4], MEMS/NEMS [5], …) with the extensive changes it creates in the properties of materials. Studies show that addition of nanoparticles to base fluids can improve their lubrication and cooling characteristics.

### 1.1 Effect of nanoparticles on reducing friction and wear

Studies show that adding nanoparticles to lubricant oils leads to creation of a lubricant or adsorbent film. This film is a soft layer with low sheer tension which prevents direct contact between surfaces and reduces friction and wear [[6], [7], [8]]. For example, rheological characteristics of diamond nanoparticles distributed in lubricant oil and their lubrication performance for carbon steel and aluminum alloys investigated using pin-disk test showed a significant reduction in wear [9].

The effect of adding Fullerene nanoparticles to mineral lubricant oil on mechanical properties investigated through surface friction temperature and friction coefficient measurement using disk-on-disk test showed that Fullerene nanoparticles cause significant reduction in friction and wear. This improvement is especially obvious at higher volume fraction of Fullerene nanoparticles added to the lubricant oil [10].

Previous studies have used different mixtures of metal nanoparticles including iron (Fe), copper (Cu) and cobalt (Co) in mineral lubricant oils. Tests results have shown that adding each of these nanoparticles leads to significant reduction of friction and wear (more than 1.5 times). Results have also shown that copper nanoparticles are more effective in reducing friction and wear compared to other nanoparticles. Tests results indicate the maximum amount of wear reduction in compounds containing copper nanoparticles which indicates the important role of copper in creation of the protective lubricant films and reducing the contact between surfaces [11].

The results of thrust-ring test show that using Al_2_O_3_/TiO_2_ nanocomposites as lubricant additives at optimal concentration of 0.1 wt% can significantly reduce friction coefficient and improve wear resistance. The average reductions in friction coefficient and wear measured using thrust-ring test are 17.61 and 23.92 %, respectively. Furthermore, the reports show that Al_2_O_3_/TiO_2_ nanocomposites have a better performance in reducing friction and improving wear resistance compared to Al_2_O_3_ or TiO_2_ nanoparticles [12].

### 1.2 Effect of nanoparticles on viscosity

Viscosity is a fluid's resistance against sheet tension, one of the most important factors when selecting a suitable lubricant for any application. Lubricant oils should be viscose enough to create a thin film between moving parts and prevent direct contact. We should not assume that less viscose oils have better lubrication than heavier oils. If lubricant oil viscosity is too low, it might not be able to fully separate metal parts (due to thinning of the lubricant film). This, in turn, results in direct contact between parts, which leads to increased temperature due to friction, and the resulting heat further reduces lubricant viscosity and increases the contact surface between metal parts. A higher amount of contact surface between metal parts, in turn, increases the temperature due to friction and can lead to local welding (grappling) of metal parts and damaging the equipment. Various studies have shown that lubricant viscosity changes greatly depend on based lubricant oil, additive particles, and their concentration.

Based on previous studies on the effect of nanoparticles on the viscosity of lubricants, viscosity increases with an increase in nanoparticle volume fraction. It decreases with an increase in temperature, especially at lower temperatures.

Previous studies have shown that the effect of nanoparticles such as copper oxides, titanium (IV) oxide, and diamond on lubrication characteristics of API-SF engine oil, including viscosity, is directly dependent on the volume fraction of nanoparticles in the lubricant. With the addition of nanoparticles, SF motor oil has a higher viscosity than base SF oil. SF lubricant containing TiO2 nanoparticles has the highest viscosity and lowest friction coefficient at 40 °C.

Investigating the effect of zinc oxide (ZnO) nanoparticles on the rheological behavior of SAE-50 lubricant oil at different temperatures has shown that these lubricants show Newtonian behavior under different sheer tensions. The maximum increase in viscosity at the highest possible nanoparticle concentration and the lowest possible temperature was 12 %. The results of viscosity measurements were also compared to previous empirical and theoretical results and showed that previous models could not predict the lubricant's viscosity resulting from the addition of ZnO nanoparticles to SAE-50 [13].

The rheological behavior of SAE40 motor oil after the addition of Al2O3-MWCNTs with different volume fractions from zero to 1 vol% at a temperature range of 25–50 °C was investigated in another study. The viscosity of lubricants was measured in the sheer tension range of 1333–13333 s-1, and the results showed that all lubricants had Newtonian behavior under all conditions. The results also showed that lubricant viscosity increases with an increase in nanoparticle volume fraction and decreases with an increase in temperature. Sensitivity analysis also showed that viscosity has low-temperature sensitivity but high sensitivity to nanoparticle volume fraction [14].

### 1.3 Effect of nanoparticles on thermal characteristics of lubricants

One study showed that effective thermal conductivity measured using the Transient hot-wire technique has increased in the presence of different nanoparticles, including single-wall carbon nanotubes (SWCNT) dispersed in ethylene glycol. Adding 0.2 vol% of nanoparticles led to a 14.8 % increase in thermal conductivity [15].

Ahmedi et al., 2013 added 0.1, 0.2, and 0.5 wt% of copper (II) oxide (CuO) nanoparticles with an average particle size smaller than 100 nm to SAE 20W50 motor oil. They investigated any changes in viscosity and thermal conductivity. Their reported results showed a direct relation between the viscosity and thermal conductivity of motor oil and its nanoparticle content, with a 3 % increase in thermal conductivity at 0.1 wt% of nanoparticle [16].

### 1.4 Effect of nanoparticles on flash point, flammability, cloud point and pour point

Another critical parameter in gas turbine operation is the flash point of the lubricant oil. Flashpoint and fire point are among the oldest physical properties measured for oils. According to the ASTM definition, a flash point is the lowest temperature at which sufficient vaporized oil is present to create a momentary flame in the presence of another flame, which extinguishes quickly. The amount of vapor present at this temperature is often insufficient to create a sustainable flame, and removing the fire from the oil's surface leads to the flame being extinguished. If the oil is heated more, the amount of vapor increases, leading to a point where a momentary external fire source can lead to a flame that burns for five consecutive seconds. This temperature is defined as the fire point. These two physical characteristics measure the flammability and volatility of the oil and define the maximum working temperature of lubricants. Any oil heated to sufficiently high temperatures becomes inflammable due to a mixture of oil vapor and oxygen from the air. This means that improving lubricant oils to increase their flash and fire points can improve the work safety of gas turbines while reducing the risks of fire and explosion due to elevated temperatures.

Cloud point is the temperature at which crystals are formed in oil products, giving them a wax-like appearance. Wax crystals start forming at the bottom of the container and appear at higher parts when temperature decreases based on cloud point.

The pour point is the minimum work temperature of lubricant oil and is the lowest temperature at which oil retains its fluid properties and flows out of the container. In most mineral-based industrial lubricants (designed for turbines, hydraulic systems, industrial systems, and vehicles), the pout point is the temperature at which paraffin molecules freeze into a white solid mass, causing the oil to lose all its fluidity.

Ahmedi et al., 2013 added 0.1, 0.2, and 0.5 wt% of copper (II) oxide (CuO) nanoparticles to SAE 20W50 motor oil and investigated any changes in pour point and fire point. Their results showed a direct relation between nanoparticle concentration and the fire point of motor oil, with the fire point increasing by 7.5 % at 0.1 wt% of the nanoparticle. Furthermore, the pour point at 0.2 wt% of nanoparticles decreased by 3.7 % and 7.4 % at other nanoparticle concentrations compared to base oil [16].

Therefore, the aim of the current study is to conduct an empirical investigation on the effect of nanoparticles in improving the characteristics of HB-80 lubricant oil. Previous studies indicate that copper (II) oxide (CuO) nanoparticles have promising results for improving lubrication properties. We also used two new nanoparticles including graphene oxide (GO) and molybdenum (VI) oxide (MoO_3_) as additives for the base lubricant and investigated the effects of these additives on lubricant properties. As previously mentioned, the HB80 oil, performs two major tasks including cooling and lubrication. In previous articles, the effect of adding nanoparticles on one of the two characteristics of cooling or lubrication has been studied. Because the addition of nanoparticles may improve some of the properties of the oil, but at the same time weaken other properties, in this research, a comprehensive study has been conducted on the effect of adding nanoparticles on all properties of oil, including density, flash and fire points, cloud and pour points, viscosity and viscosity indicator, thermal conductivity coefficient, friction coefficient and anti-wear performance of the lubricant.

The main reason for the current study is to increase the shelf life of gas turbine parts by adding nanoparticles to lubricant oils in order to improve thermal conductivity and reducing friction in moving parts of the turbine and compressor. This study for the first time investigates the effect of copper (II) oxide (CuO), graphene oxide (GO) and molybdenum (VI) oxide (MoO_3_) on HB-80 lubricant.

## 2 Materials and methods

### 2.1 Base oil

The aim of this study is to investigate the effect of nanoparticles on HB-80 oil produced by Iranol Co. The properties of the base oil provided by the manufacturer are presented in Table 1.

**Table 1:** Characteristics of HB-80 industrial oil.

Parameter	Test method	Test conditions	Unit	Quantity
Kinematic viscosity	D-455	40 °C	Centistokes (cSt)	46
Viscosity Index	D-2270	40 & 100 °C	–	95
Flash point	D-92	–	%C	205
Pour point	D-97	–	%C	−6
Density	D-1298	15 °C	Kg/m^3^	875
Acid number	D-664	–	mg KOH/g	15/0
Copper corrosion	D-130	100 °C	–	Class 1a
Getting out of the water	IP 19	–	Secs	300

### 2.2 Nanoparticles

This study uses copper (II) oxide nanoparticles due to their performance in improving lubrication, as well as two new nanoparticles, including graphene oxide (GO) and molybdenum (VI) oxide (MoO3).

Copper (II) oxide nanoparticles have desirable characteristics such as high thermal conductivity, suitable corrosion resistance, high flexibility, and high strength. Studies show that copper (II) nanoparticles create a fragile and self-repairing lubricant film between surfaces, preventing direct contact between surfaces and decreasing friction and adhesion. This leads to a significant reduction in friction and wear, leading to widespread CuO use in many studies. Graphene oxide has a single-layer carbon structure with a significant active surface and is a compressed and honeycomb structure. The presence of epoxide, carboxyl, and hydroxyl groups on the surface of graphene oxide and its high specific surface area has gained the attention of many researchers who have found many uses for GO, including in novel technologies. Graphene oxide creates a slippery surface and is therefore used to reduce wear and friction. The other nanoparticle, molybdenum (VI) oxide (MoO3), has been used for the first time in lubrication studies. This nanoparticle is a solid with a rare, layered crystalline structure in which each molybdenum atom is surrounded by an octagon of oxygen atoms (Orthorhombic structure). Due to its layered structure, easy bonding of Mo atoms, and high thermal conductivity, this nanoparticle is used in various applications. CuO, GO, and MoO3 nanoparticles used in the current study with properties shown in Table 2 were acquired from US Research Nano company.

**Table 2:** Characteristics of CuO, GO and MoO_3_ nanoparticles.

Nanoparticle Parameter	copper oxide	graphene oxide	molybdenum oxide
Code(CAS NO)	1317-38-0	7782-42-5	1313-27-5
Purity(%)	99.9	99.2	99.9
Color	Black	Gray-Brown	Dark red
Average particle size(nm)	40	–	13–80
Special level(m^2^/g)	20	100	–
Particle density (g/cm^3^)	6.4	2.1	4.69

**Fig. 1:**
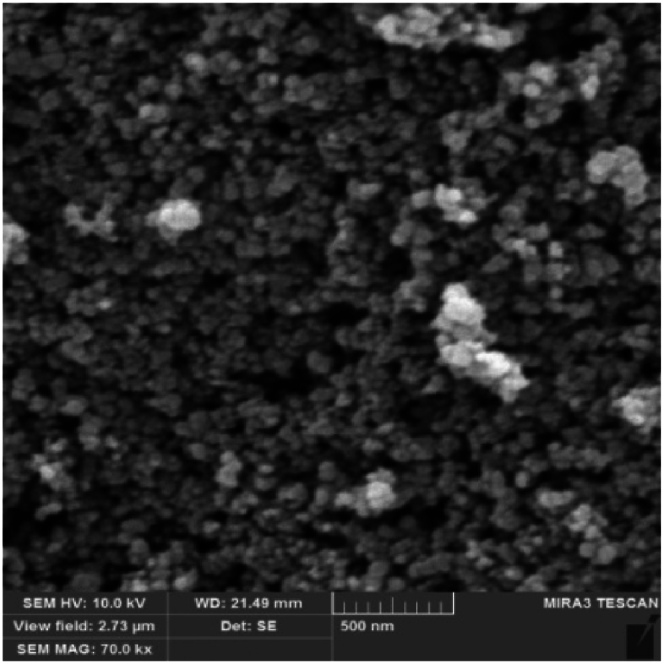
SEM image of CuO nanoparticles.

**Fig. 2:**
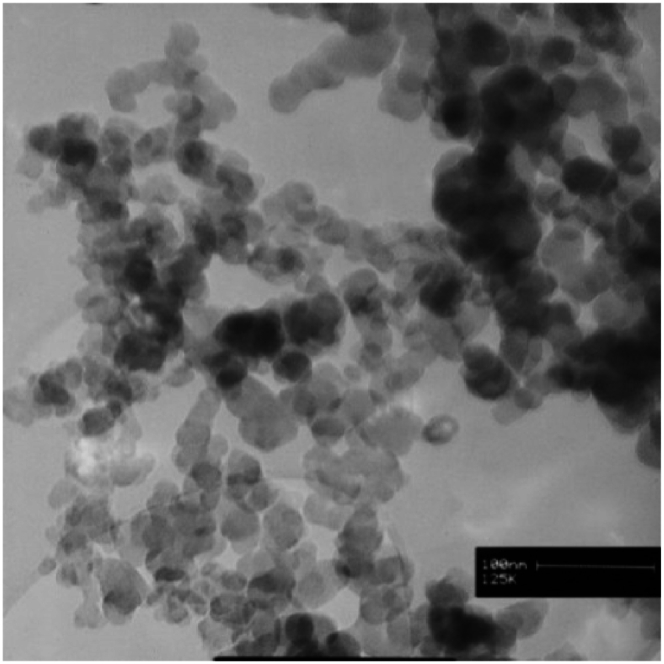
SEM image of MoO_3_ nanoparticles.

**Fig. 3:**
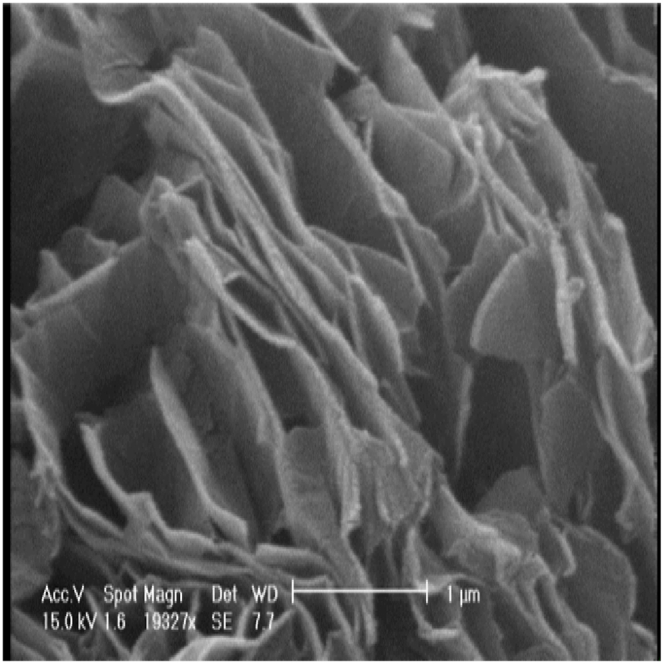
SEM image of GO nanoparticles.

SEM and TEM images offered by the manufacturer of nanoparticles were deemed sufficient for evaluating the morphology of the particles. SEM images of nanoparticles (Fig. 1, Fig. 2, Fig. 3) show that copper (II) oxide and molybdenum (VI) oxide nanoparticles have spherical morphology, while graphene oxide nanoparticles are sheets with thickness in the nanometer range. Multi-layered graphene oxide nanoparticles were the type used in this study.

**Fig. 4:**
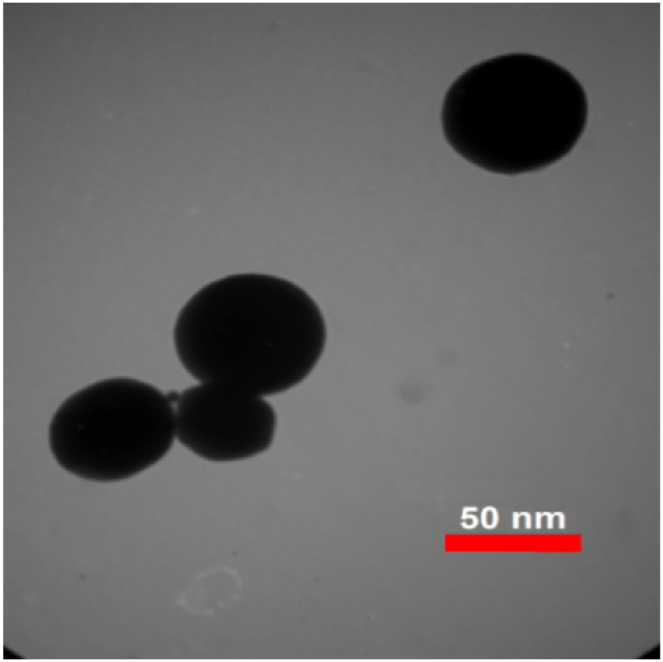
TEM image of CuO nanoparticles.

**Fig. 5:**
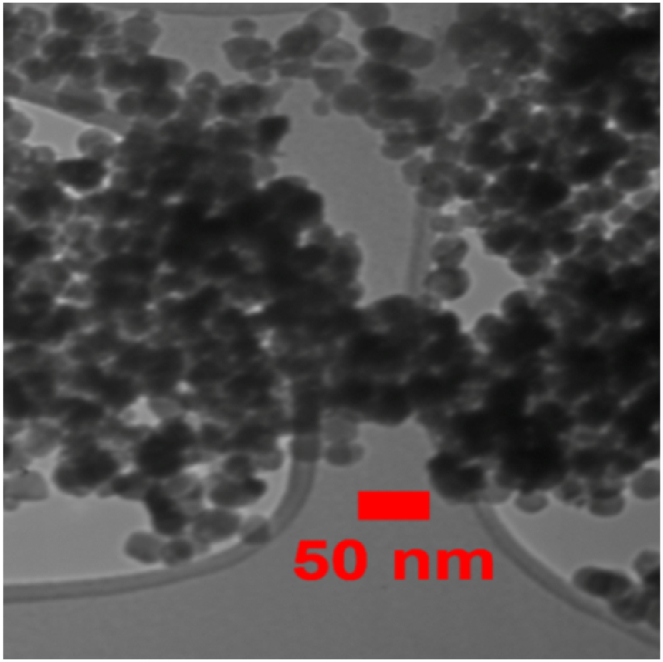
TEM image of MoO_3_ nanoparticles.

**Fig. 6:**
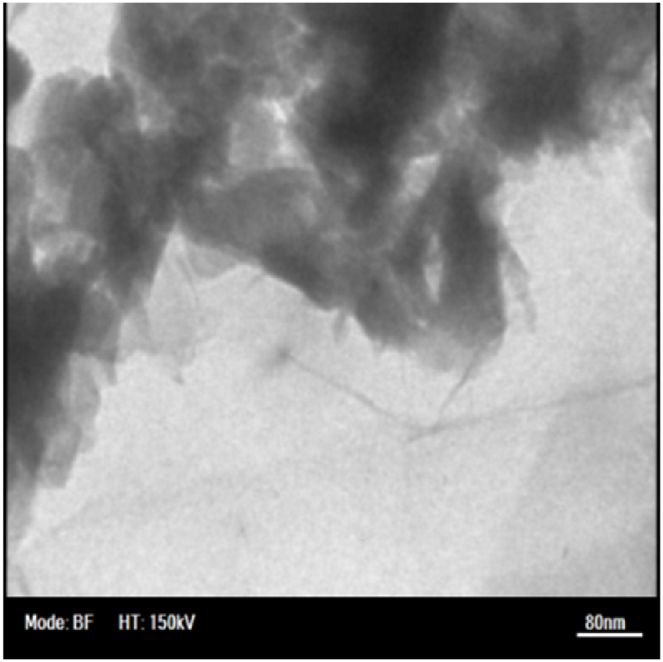
TEM image of GO nanoparticles.

TEM images presented in Fig. 4, Fig. 5, Fig. 6 were used to determine the average size of nanoparticles. According to these images, copper (II) oxide and molybdenum (VI) oxide nanoparticles have an average size of around 50 nm. However, in case of graphene oxide particles, sheet thickness is around 15 nm while other dimensions are in micrometer range.

### 2.3 Preparation of nanofluid

**Fig. 7:**
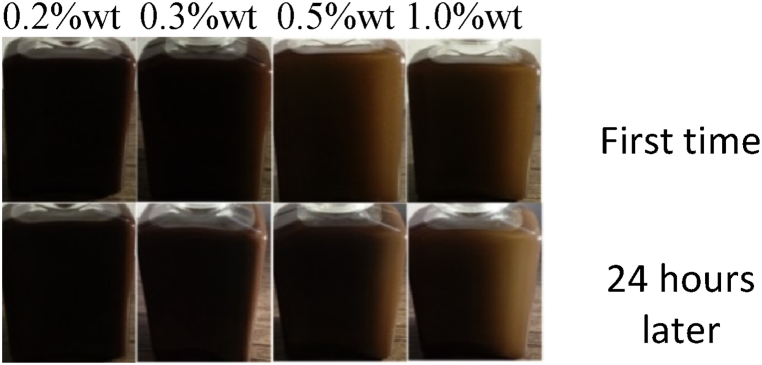
Dispersion of CuO nanoparticles.

**Fig. 8:**
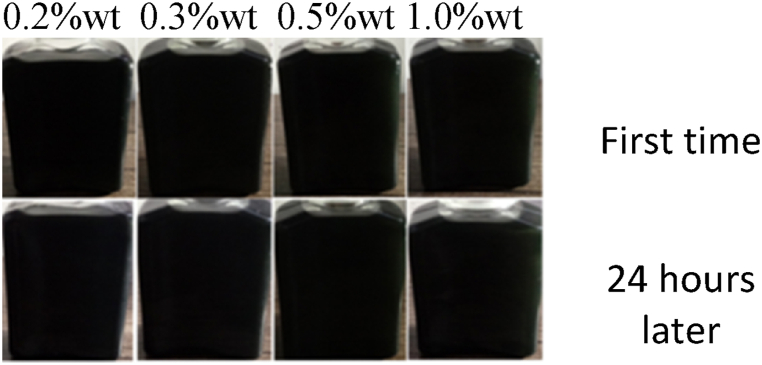
Dispersion of GO nanoparticles.

**Fig. 9:**
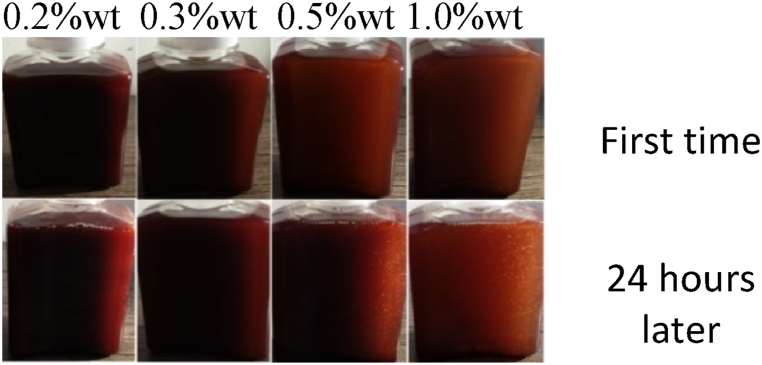
Dispersion of MoO_3_ nanoparticles.

Nano-lubricants are created by dispersing a desired concentration of nanoparticles in base lubricant oil. In the current study, nanofluids were prepared using 0.2, 0.3, 0.5 and 1 wt% of CuO, MoO_3_ and GO nanoparticles. To this end, magnetic stirring for 6 h was used to disperse nanoparticles in HB-80 base oil. Span 80 was also used as dispersant. Fig. 7, Fig. 8, Fig. 9 show the images of nanofluid immediately after preparation and 24 h later. As can be seen, nanofluids have acceptable stability.

### 2.4 Measurement of lubricants’ properties

This study measured the density of lubricant samples with high precision at 20 °C. The hydrometer used in this test was a type 850–900 hydrometer from Brannan CO, UK, with a maximum measurement error of 0.0006, maximum precision of 0.001, and length of 270 mm.

**Fig. 10:**
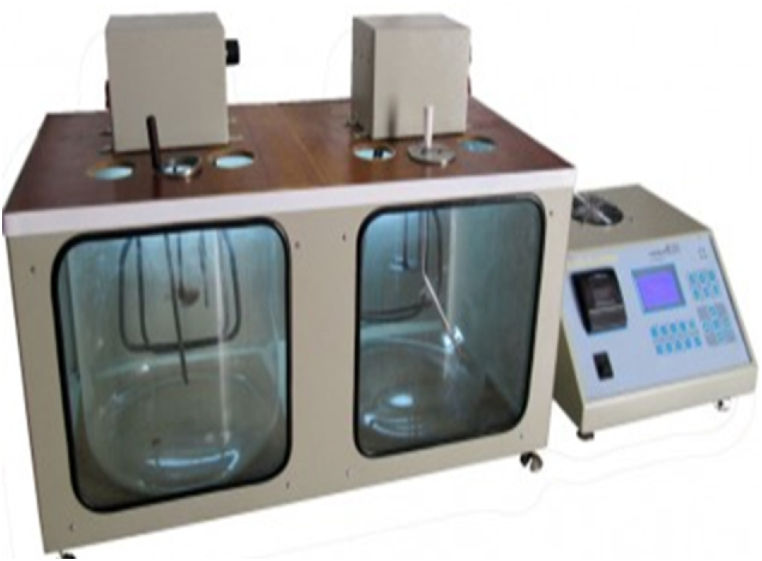
Twin viscosity bath instrument made by Iranian Experiment Tool Co.

The kinematic viscosity of nanofluids was measured at two temperatures of 40 and 100 °C based on ASTM D445 standard using a twin viscosity bath instrument made by Iranian Experiment Tool Co. and reported in Centistokes (CST). The instrument can measure fluid viscosity in the range of 6–100 cst with a precision of 0.1 cst (Fig. 10).

Another important property of lubricants is their viscosity indicator (VI). This indicator is a dimensionless number which shows temperature dependence of kinematic viscosity. Viscosity indicator is calculated according to ASTM D2270 standard using equation (1).

(1) $$\text{VI}=100\frac{L-U}{L-H}$$

where U is the kinematic viscosity of the oil at 40 °C (104 °F). L and H are viscosity at 40 °C for reference oils with VI of 0 and 100, respectively, which have similar viscosity to the oil being tested at 100 °C. Values for L and H can be extracted form ASTM D2270 standard.

**Fig. 11:**
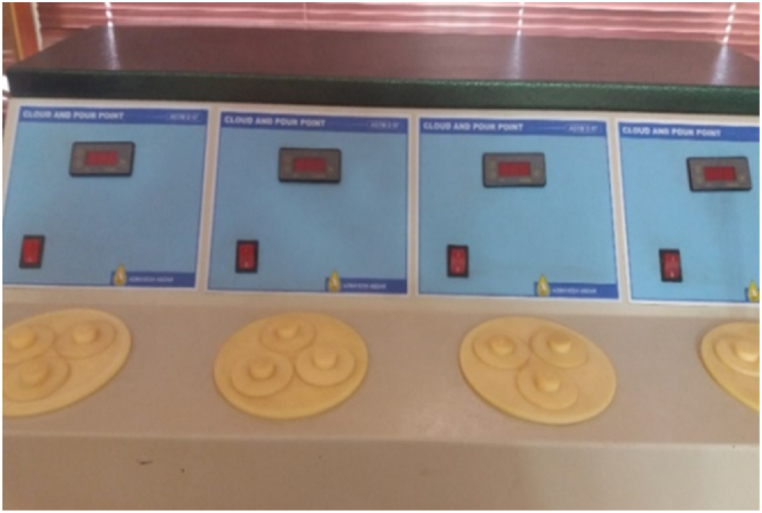
Freezing instrument of Iranian Experiment Tool Co.

Cloud point and pour point of lubricants were measured using ASTM D2500 and ASTM D97 standards with Freezing instrument of Iranian Experiment Tool Co. with precision of 0.1 °C (Fig. 11).

Flash point and fire point of Nano-lubricants were measured using ASTM D92 standard and Cleveland Open flash point instrument of Iranian Experiment Tool Co. with precision of 0.1 °C.

In order to measure thermal conductivity coefficient, a Thermal analyzer instrument (Decagon Company, KD2) was used. This instrument uses a temperature control bath with precision of 0.1 °C to control fluid temperatures. Lubricant samples were synthesized and put into the bath for 30 min in order to achieve temperature stability before measuring their thermal conductivity coefficient. Measuring thermal conductivity coefficient for each lubricant sample was carried out in 10 steps. Due to the mechanism used in measurement of thermal conductivity coefficient with unusable hot wire, each step of measurement causes the fluid to lose its temperature stability. As a result, 5 min was allowed to lapse between two consecutive measurements steps.

**Fig. 12:**
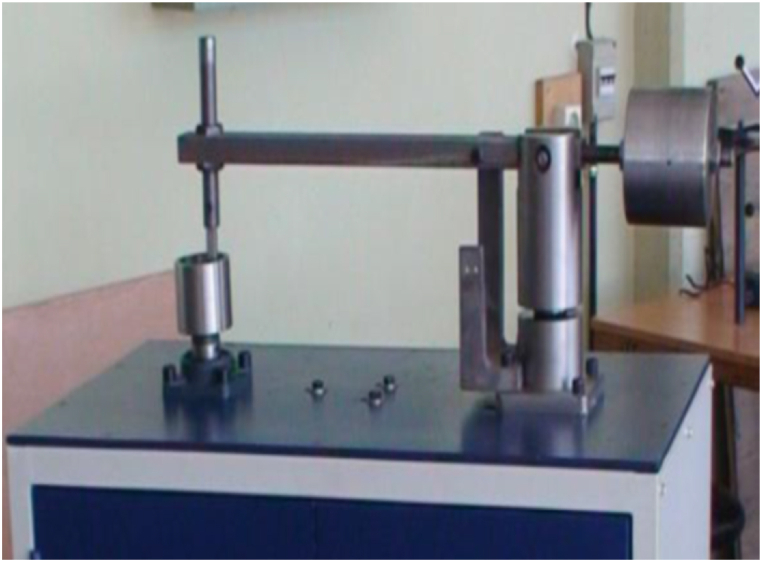
Pin-on-disk instrument.

Several methods are used to investigate anti-wear and antifriction performance of lubricants, one of the most common of which is pin-on-disk test. Pin-on-disk instrument is used to predict the lubricant behavior of engineering materials and alloys under working conditions. This instrument can predict lubricant performance by measuring wear and friction coefficient. This instrument measured anti-wear and antifriction performance of lubricants at room temperature according to ASTM G99 standard. The pin and disc are from T5 60 HRC hardness and stainless steel (360), respectively. All of the experiments were repeated 3 times and the average values were reported. Test setup and test conditions are shown in Fig. 12 and Table 3.

**Table 3:** Conditions of pin-on-disk test.

Parameter	Unit	Quantity
Pin diameter	mm	8
Disk diameter	mm	45
Diameter of abrasion path	mm	40
Spin speed	rpm	150
Vertical force	N	150
Humidity	–	25 %
Friction measurement precision	N	0.049
Wear measurement precision	mg	0.1

## 3 Results and discussion

### 3.1 Effect of nanoparticles on density

The results of density measurements for HB-80 base lubricant oil as well as lubricants with 0.2, 0.3, 0.5 and 1 wt% of CuO, MoO_3_ and GO are shown in Table 4. As we predicted, the effect of nanoparticles on lubricant density was negligible due to small weight percent of nanoparticles. The results also show that increase in nanoparticle concentration increases the density of the lubricant oil. Graphene oxide nanoparticles have the lowest density and therefore have the lowest amount of effect on lubricant density. The reason is that density of a mixture depends on the density and mass fraction of its components. Although nanoparticles have a higher density than lubricant oil, their low weight fraction means that their effect on total density of mixture is small.

**Table 4:** Density of different lubricants with different weight fraction of nanoparticles.

Nanoparticle	Density (g/cm^3^)				
	Concentration of nanoparticles (wt%)				
	Bace oil (0.0)	0.2	0.3	0.5	1.0
copper oxide	0.876	0.877	0.878	0.880	0.884
graphene oxide	0.876	0.877	0.877	0.878	0.881
molybdenum oxide	0.876	0.877	0.878	0.879	0.883

### 3.2 Effect of nanoparticles on viscosity and viscosity index

**Fig. 13:**
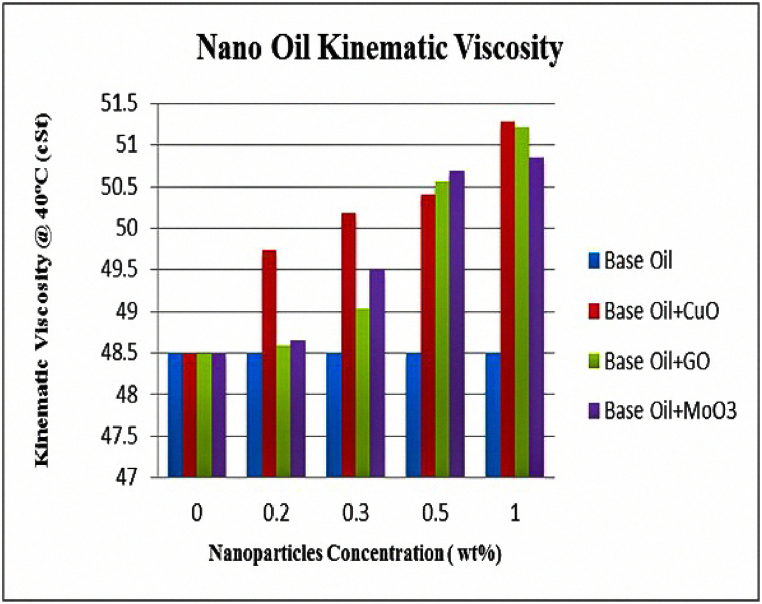
Kinematic viscosity at 40 °C.

**Fig. 14:**
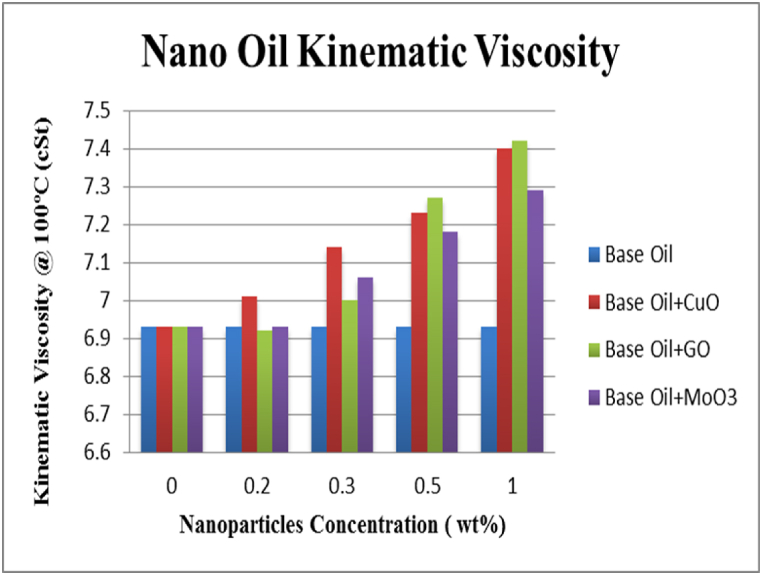
Kinematic viscosity at 100 °C.

Kinematic viscosity of the lubricants at different concentrations of nanoparticles at two temperatures of 40 and 100 °C are shown in Fig. 13, Fig. 14. The results show the increase in viscosity due to adding nanoparticles. Increase in viscosity is also reported in previous studies [13,14]. These results indicate that the highest amount of increase for CuO, GO and MoO_3_ nanoparticles is seen at 100 °C for 1 wt% of nanoparticle and is equal to 6.78, 7.07 and 5.19, respectively. The results also indicate that there is a direct and positive correlation between weight fraction of nanoparticles and viscosity of the lubricant. One interesting point is that temperature greatly affects viscosity and increasing temperature from 40 to 100 °C reduces viscosity by more than 85 %. Results presented in Table 5 show that increase in nanoparticle content increases viscosity index (VI) of the lubricant and reduces viscosity changes with increase in temperature due to improving thermal stability of the lubricant. The highest amount of increase in VI for CuO, GO and MoO_3_ nanoparticles is seen at 1 wt% and is equal to 8.60, 8.70 and 6.53 %, respectively. Viscosity dependence of temperature is measured using VI. Decrease in temperature leads to an increase in viscosity and if temperature increases, viscosity will decrease. This fact is more important in cases where lubricant viscosity changes greatly with temperature. VI is a numerical value showing viscosity changes of oils with temperature and a larger VI means that viscosity changes due to temperature are smaller. Usually polymers with high molecular weights are used to increase VI of oils which also increase the viscosity itself and might cause problems in lubrication.

**Table 5:** Kinematic viscosity and VI of different nanofluids at different nanoparticle concentrations at 40 and 100 °C.

Type of lubricant	Concentration of nanoparticles	Viscosity at 40 °C (cSt)	Increase rate (%)	Viscosity at 100 °C (cSt)	Increase rate (%)	Viscosity Index(%)	Increase rate (%)
Bace oil	0.0	48.49	–	6.93	–	96.5	–
copper oxide + Bace oil	0.2	49.73	2.55	7.01	1.15	96.1	−0.41
	0.3	50.17	3.46	7.14	3.03	98.2	1.76
	0.5	50.40	3.93	7.23	4.33	100.9	4.56
	1.0	51.27	5.73	7.40	6.78	104.8	8.60
graphene oxide + Bace oil	0.2	48.58	0.19	6.92	−0.14	96.2	−0.31
	0.3	49.02	1.09	7.00	1.01	98.5	2.07
	0.5	50.56	4.27	7.27	4.91	103.7	7.46
	1.0	51.22	5.63	7.42	7.07	104.9	8.70
molybdenum oxide + Bace oil	0.2	48.65	0.33	6.93	0.0	95.9	−0.62
	0.3	49.51	2.10	7.06	1.88	100.3	3.43
	0.5	50.68	4.52	7.18	3.61	100.9	4.56
	1.0	50.85	4.87	7.29	5.19	102.8	6.53

### 3.3 Effect of nanoparticles on flash and fire points

**Fig. 15:**
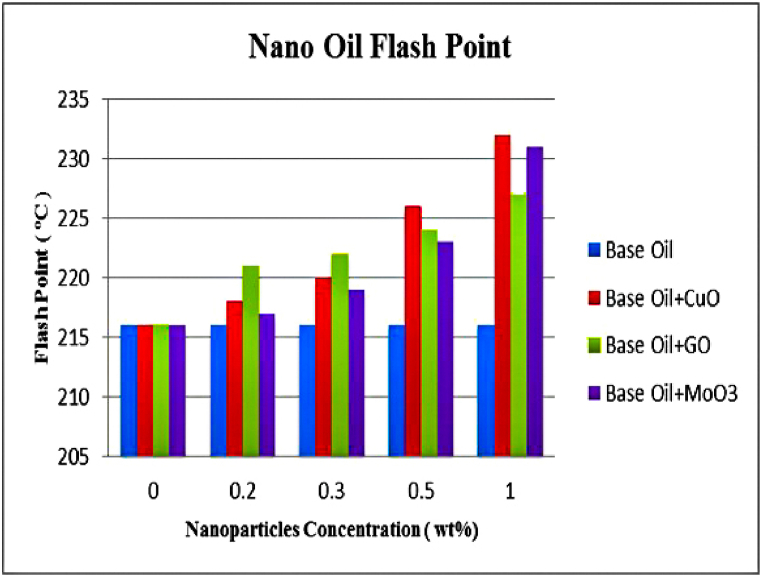
Flash point versus concentration.

**Fig. 16:**
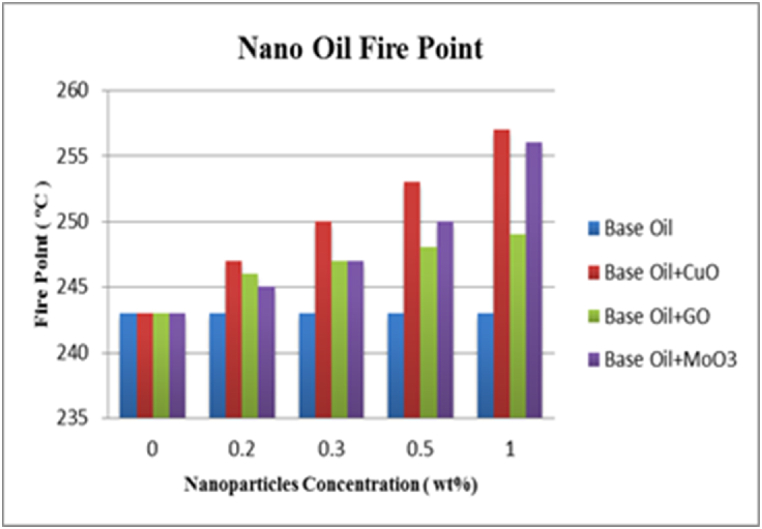
Fire point versus concentration.

As mentioned previously, flash and fire points indicate the maximum working temperature of lubricants. The results of measurements for flash and fire points of HB-80 base oil and mixtures containing CuO, GO and MoO_3_ nanoparticles at 0.2, 0.3, 0.5 and 1 wt% are shown in Fig. 15, Fig. 16. The results undoubtedly show that adding nanoparticles to the base oil increases its flash and fire points and this increase is larger at higher weight fractions of nanoparticle. Due to their high thermal conductivity, CuO nanoparticles at 1.0 wt% create the largest increase in flash and fire points by 7.41 and 5.76 %. With increase in the concentration of nanoparticles, thermal conductivity of lubricant improves which increase its flame resistance. Since all three nanoparticles improve thermal conductivity of the lubricant, this increasing trend can be seen in all three mixtures. However, this effect is smaller for graphene oxide nanoparticles which is due to lower thermal conductivity of this nanoparticle. As mentioned before, higher fire point means that it is somewhat possible to use the lubricant at higher temperatures but normally lubricant oils with higher fire points contain hydrocarbons with high molecular weights and therefore high viscosities. Very high viscosity is a disadvantage in lubricants and can lead to serious problems in lubricated systems. However, as seen in Fig. 16, nanoparticles significantly increase fire point of lubricants compared to the base oil. On the other hand, data presented in Table 5 shows that compared to the increased in fire point, the increase in viscosity is negligible. In general, increased flame resistance of lubricants is due to increased thermal conductivity of the oil. This increase in fire point is one of the positive effects of nanoparticles on properties of base lubricant oil.

### 3.4 Effect of nanoparticles on cloud and pour points

**Fig. 17:**
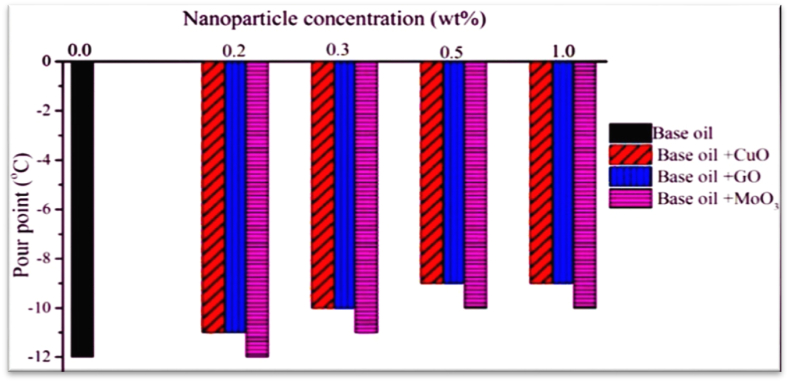
Pour point versus concentration.

Table 6 and Fig. 17 show the results of cloud and pour point measurements for base oil and lubricants containing CuO, GO and MoO_3_ nanoparticles at 0.2, 0.3, 0.5 and 1 wt%. At nanoparticle concentrations higher than 0.2 wt%, cloud point can't be determined due to darkening of the oil. However, as can be seen in Fig. 17, adding nanoparticles increases pour point of lubricants. Copper (II) oxide and graphene oxide nanoparticles show similar behaviors and increase pour point by the same amount with biggest increase being 25 % at nanoparticle concentration of 1 wt% while molybdenum oxide nanoparticles have a smaller effect on pour point. Although this increase in pour point has no significant effects on lubrication range of the oil, the best nanoparticle concentration is 0.2 wt% which causes no significant changes in cloud and pour points while improving other parameters of the oil.

**Table 6:** Cloud point of different lubricants with different concentrations of nanoparticles.

Nanoparticle	Cloud points (%C)				
	Concentration of nanoparticles (wt%)				
	Bace oil (0.0)	0.2	0.3	0.5	1.0
copper oxide	−8.0	−6.0	–	–	–
graphene oxide	−8.0	−7.0	–	–	–
molybdenum oxide	−8.0	−7.0	–	–	–

**Fig. 18:**
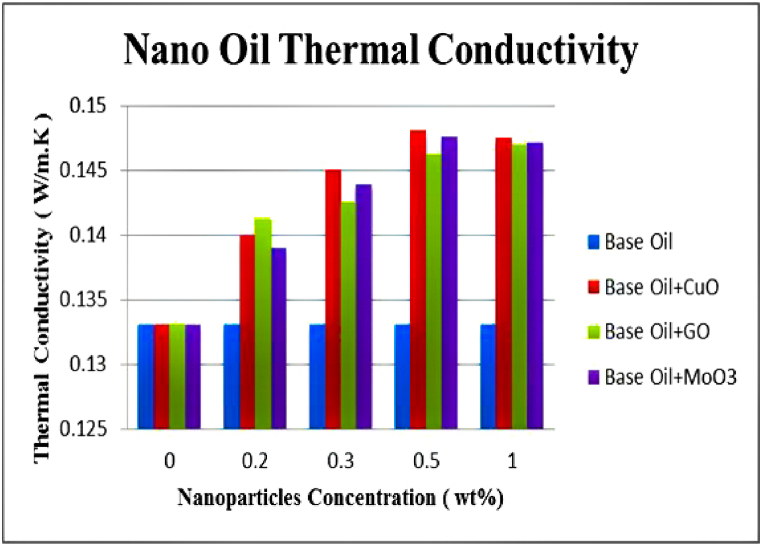
Thermal conductivity coefficients versus concentration.

### 3.5 Effect of nanoparticles on thermal conductivity

- Thermal conductivity coefficients of pure base oil and oils containing CuO, GO and MoO_3_ nanoparticles at 0.2, 0.3, 0.5 and 1 wt% are shown in Fig. 18. The results clearly indicate that adding nanoparticles improves thermal conductivity of lubricants. Similar trends are reported by Ahmadi et al. [16].These results show that the effect of all three nanoparticles is almost identical and increases thermal conductivity coefficient by the same amount. However, at high nanoparticle contents, thermal conductivity remains almost unchanged which can be accumulation and instability of nanoparticles at high concentrations. Highest increase in thermal conductivity coefficient is seen at 1 wt% of CuO with 11.3 % increase compared to the base oil. This increase is due to high thermal conductivity of this nanoparticle. Increase in thermal conductivity coefficient can improve heat transfer in gas turbines and compressors. Increase in thermal conductivity coefficient of lubricants increases heat transfer from gas turbine and compressor bearings to air coolers of the oil. Therefore, it will be possible to remove excess heat from the system with a lower flow of the oil. This reduction in necessary oil flow reduces the power needed for the oil pump. Furthermore, in the oil cooling system, it will be possible to conduct the necessary heat transfer with a smaller number of cooling units which in turn leads to lower consumption of electrical energy by electromotors used to operate cooling fans.

### 3.6 Effect of nanoparticles on wear

**Fig. 19:**
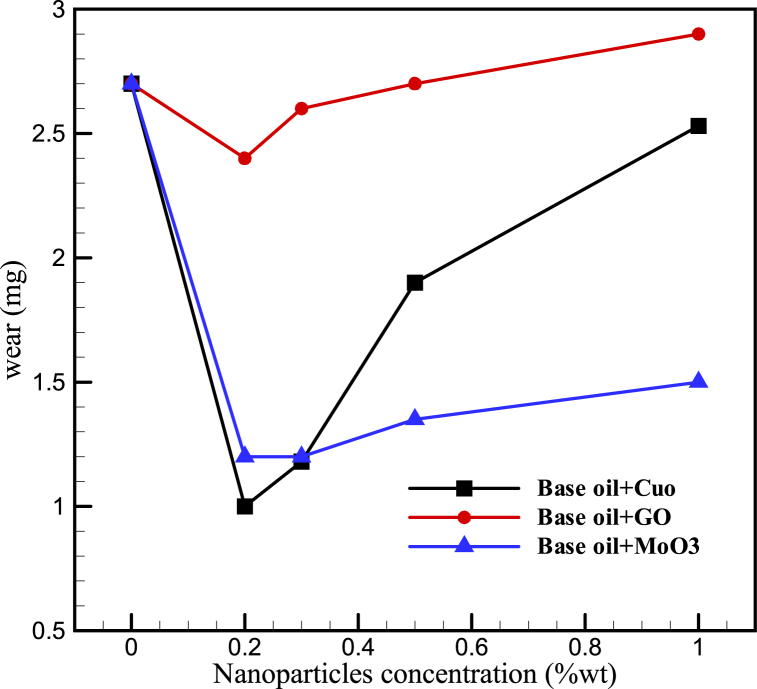
Wear loss in parts while using base oil and oil containing CuO, GO, and MoO_3_ at concentrations of 0.2, 0.3, 0.5 and 1 wt%.

Current study investigates the effect of adding CuO, GO and MoO_3_ nanoparticles on anti-wear characteristics of HB-80 lubricant oil. Pin-on-disk test was carried out for pure lubricant oil as well as mixtures containing 0.2, 0.3, 0.5 and 1 wt% of nanoparticles the results of which are shown in Fig. 19. The figure shows the wear of the whole system (disk and counter body). As can be seen, with increase in nanoparticle concentration, wear first decreases and then increases with lowest wear being at 0.2 wt% of CuO nanoparticle which is 62.96 % lower than the base oil. Wear of parts not only reduce their lifetime but also creates residues which reduces the quality of lubricant. This means that anti-wear characteristics have a significant effect on lubrication performance of oils. According to various studies, there are several reasons for improvement observed in compressibility and anti-wear properties of lubricants after addition of nanoparticles [6,7,17].

1. Nanoparticles act similar to ball bearings between two friction surfaces and reduce contact between parts
2. Nanoparticles cover rough surfaces and crease a lubricant and protective layer or film between two surfaces
3. Added nanoparticles have a restorative effect; precipitating on the surfaces and filling the scratches therefore compensating for the lost mass and reducing surface roughness

The reason for increased wear at higher concentration of nanoparticles can be instability and accumulation of particles and destruction of the protective lubricant film. Previous studies also report a similar phenomenon [ [4,11]]. Furthermore, these results show that graphene oxide nanoparticles have a lower performance in reducing wear compared to copper (II) oxide and molybdenum (VI) oxide particles which can be due to their lower ability in creating protective lubricant layer. Previous studies have shown that copper (II) oxide nanoparticles have a high ability for creating a lubricant layer and reducing wear. This fact is observed in the current results where copper (II) oxide has a good performance at lower concentrations. However, at higher nanoparticle concentrations, molybdenum (VI) oxide particles show a better performance.

### 3.7 Effect of nanoparticles on friction coefficient

**Fig. 20:**
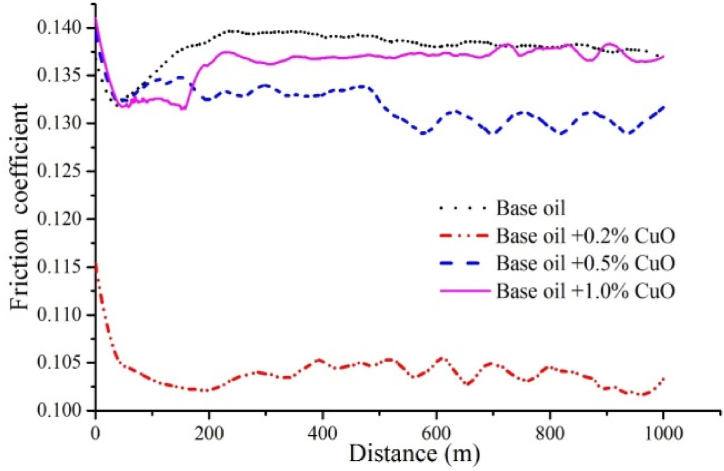
Friction coefficient based on distance travelled for base oil and oil containing copper (II) oxide at concentrations of 0.2, 0.5 and 1 wt%.

**Fig. 21:**
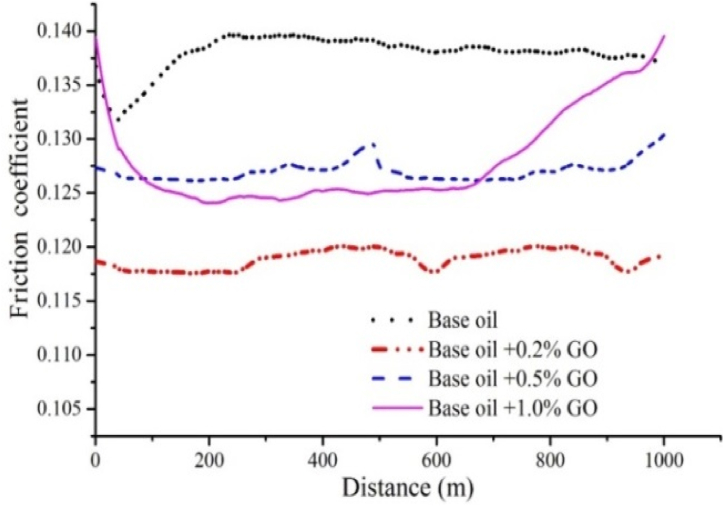
Friction coefficient based on distance travelled for base oil and oil containing graphene oxide at concentrations of 0.2, 0.5 and 1 wt%.

**Fig. 22:**
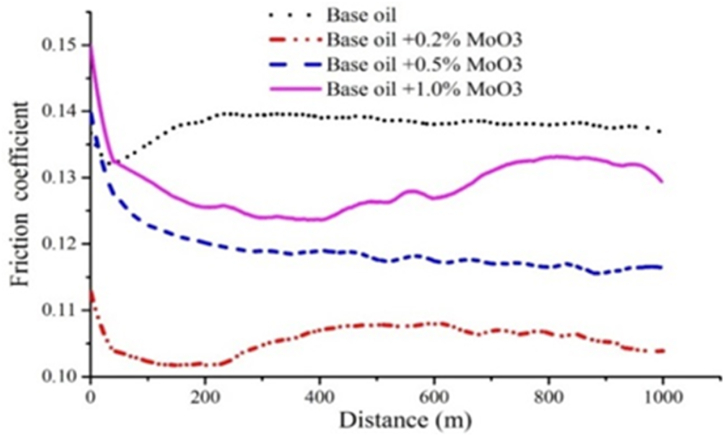
Friction coefficient based on distance travelled for base oil and oil containing molybdenum (VI) oxide at concentrations of 0.2, 0.5 and 1 wt%.

**Fig. 23:**
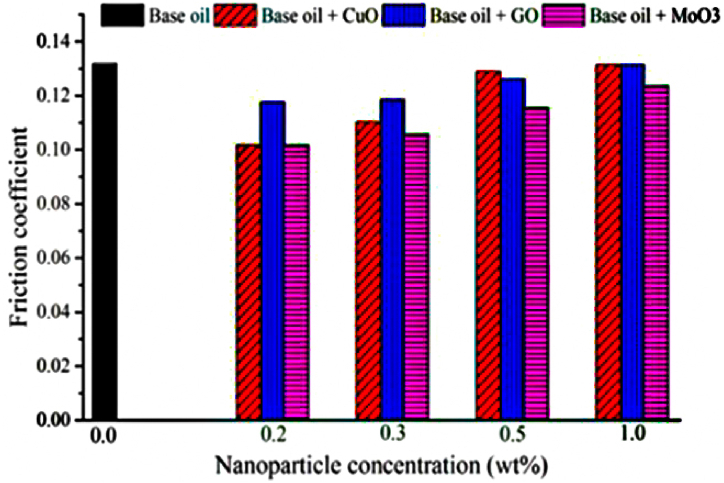
Friction coefficient for base oil and oil containing CuO, GO and MoO_3_ at concentrations of 0.2, 0.3, 0.5 and 1 wt%.

The results of friction coefficient based on distance travelled (calculated by converting number of rotations to distance travelled) for base oil as well as oils containing 0.2, 0.5 and 1 wt% of CuO, GO and MoO_3_ nanoparticles are shown in Fig. 20, Fig. 21, Fig. 22. In order to provide a clearer view, the results for each nanoparticle are shown in a separate graph. Furthermore, due to similarity of results for 0.2 and 0.3 wt%, the results for 0.3 wt% are eliminated. Additionally, and in accordance with previous studies, lowest friction coefficient for each graph is calculated and is shown in Fig. 23. The results obtained for friction coefficient are similar to the results of wear test. This is in agreement with the results of ref. [12]. At low concentrations of nanoparticle, a protective lubricant film is formed due to higher stability of nanoparticles which reduces contact and friction coefficient. Copper (II) oxide and molybdenum (VI) oxide particles show better performance compared to graphene oxide particles and the results presented in Fig. 23 show that the largest decrease in friction coefficient is at 0.2 wt% of CuO and MoO_3_ with 22.86 % and 19.02 % decrease, respectively.

## 4 Conclusion

The main goal of the current study was to conduct an empirical investigation on ability and performance of copper (II) oxide (CuO), graphene oxide (GO) and molybdenum (VI) oxide (MoO_3_) nanoparticles for improving performance of HB-80 lubricant oil and reducing energy cost of equipment. Nano-lubricants containing these nanoparticles with weight percentage of 0.2, 0.3, 0.5 and 1 % were prepared and their important characteristics including density, flash and fire points, cloud and pour points, viscosity and viscosity indicator, thermal conductivity coefficient, friction coefficient and anti-wear performance were measured using various empirical tests.

The results of these experiments can be summarized as follows.

- Density and viscosity of lubricants increases with addition of nanoparticles proportional to the weight fraction.
- The effect of addition of nanoparticles on viscosity indicator, is higher than its effect on viscosity. Increase in nanoparticle concentration leads to increase in viscosity indicator. Variations of viscosity and viscosity indicator are almost identical for CuO, GO and MoO_3_.
- Flash point and fire point increase with addition of nanoparticles. CuO nanoparticles with weight fraction of 1 % caused the largest increase in flash and fire points with 7.41 and 5.76 % increase, respectively.
- Copper (II) oxide and graphene oxide nanoparticles both have a similar trend and increase pour point by similar amounts while molybdenum (VI) oxide nanoparticles have a smaller effect on pour point. However, this increase in pour point has no significant effects on lubricant's performance.
- Addition of nanoparticles increases thermal conductivity of the lubricants Largest increase in thermal conductivity was equal to 11.3 % increase compared to the base oil for 1 wt% of CuO nanoparticles which is due to high thermal conductivity of CuO nanoparticles.
- Addition of nanoparticles to lubricant oil creates a protective lubricant film thus reducing friction coefficient and wear. This is especially obvious for lower concentrations of nanoparticles which leads to formation of a more stable protective film. Copper (II) oxide nanoparticles with weight fraction of 0.2 % have the best performance, reducing friction coefficient and wear by 22.86 and 62.96 %, respectively.

Finally, these results indicate that copper (II) oxide nanoparticles have the best performance in improving lubrication, anti-wear, friction and thermal conductivity properties of the lubricant oil with 0.2 wt% of CuO nanoparticles being the optimum concentration.

## CRediT authorship contribution statement

**Shahram Karimi:** Writing – original draft, Data curation. **Amir Homayoon Meghdadi Isfahani:** Writing – review & editing, Supervision. **Masoud Afrand:** Methodology, Investigation. **Mohammad Akbari:** Writing – review & editing.

## Declaration of competing interest

The authors declare that they have no known competing financial interests or personal relationships that could have appeared to influence the work reported in this paper.
